# Integrative Approaches for Studying Mitochondrial and Nuclear Genome Co-evolution in Oxidative Phosphorylation

**DOI:** 10.3389/fgene.2017.00025

**Published:** 2017-03-03

**Authors:** Paul Sunnucks, Hernán E. Morales, Annika M. Lamb, Alexandra Pavlova, Chris Greening

**Affiliations:** ^1^School of Biological Sciences, Monash University, ClaytonVIC, Australia; ^2^Department of Marine Sciences, University of GothenburgGothenburg, Sweden

**Keywords:** mitochondrial, nuclear, mitonuclear, oxidative phosphorylation, OXPHOS, co-evolution, genome architecture

## Abstract

In animals, interactions among gene products of mitochondrial and nuclear genomes (mitonuclear interactions) are of profound fitness, evolutionary, and ecological significance. Most fundamentally, the oxidative phosphorylation (OXPHOS) complexes responsible for cellular bioenergetics are formed by the direct interactions of 13 mitochondrial-encoded and ∼80 nuclear-encoded protein subunits in most animals. It is expected that organisms will develop genomic architecture that facilitates co-adaptation of these mitonuclear interactions and enhances biochemical efficiency of OXPHOS complexes. In this perspective, we present principles and approaches to understanding the co-evolution of these interactions, with a novel focus on how genomic architecture might facilitate it. We advocate that recent interdisciplinary advances assist in the consolidation of links between genotype and phenotype. For example, advances in genomics allow us to unravel signatures of selection in mitochondrial and nuclear OXPHOS genes at population-relevant scales, while newly published complete atomic-resolution structures of the OXPHOS machinery enable more robust predictions of how these genes interact epistatically and co-evolutionarily. We use three case studies to show how integrative approaches have improved the understanding of mitonuclear interactions in OXPHOS, namely those driving high-altitude adaptation in bar-headed geese, allopatric population divergence in *Tigriopus californicus* copepods, and the genome architecture of nuclear genes coding for mitochondrial functions in the eastern yellow robin.

## Introduction

Rapid improvements in genomics hold much promise in advancing one of the most important but demanding tasks in evolutionary biology: establishing genotype-to-phenotype links for features of organisms that are important in adaptation and speciation (Savolainen et al., 2013; Seehausen et al., 2014). The main challenge is that fitness-conferring characteristics in complex organisms are typically quantitative traits, controlled by many loci with small individual effect sizes (Mackay et al., 2009). This is compounded by the astronomical numbers of both meaningful gene interactions and spurious correlations that arise from population structure and history. Accordingly, adaptation can be implicated in species evolution only when disentangled from population history (Hoban et al., 2016). Identifying genotype-to-phenotype links of complex traits can be made more tractable by focussing on genomic variation expected to bestow major fitness differences based on prior knowledge. If such predictions are consistent with population genomic analyses, this will increase confidence that the candidate genes and mechanisms are true positives worthy of the demanding empirical investigations in wild populations needed to test them (Cheviron et al., 2014; Gompert et al., 2014; Egan et al., 2015).

An excellent opportunity to study the interplay between population biology and genome architecture is presented by interactions between mitochondrial proteins encoded by genes of the mitochondrial and nuclear genomes. Such mitonuclear interactions are required for fundamental physiological processes such as cellular respiration (Bar-Yaacov et al., 2012) and thus influence processes at multiple levels of biological organization: cellular function, organismal fitness, and ecosystem processes (Dowling et al., 2008; Wolff et al., 2014; Latorre-Pellicer et al., 2016). Moreover, these interactions are so central to evolutionary and ecological processes, including adaptation and speciation, that the term ‘mitonuclear ecology’ was recently proposed for their study (Hill, 2015, 2016).

While we have an incomplete understanding of most mitonuclear interactions (Pagliarini et al., 2008), we have a rich knowledge of a fundamental subset of them: those that form the core protein complexes responsible for oxidative phosphorylation (OXPHOS) (Rand et al., 2004; Gershoni et al., 2009). This essential system is responsible for the availability of nearly all cellular energy in eukaryotes, and thus through metabolic, trophic and thermal biology, at some level underpins virtually all eukaryotic ecological and evolutionary phenomena (Rand et al., 2004). These interactions are amenable to experimental investigation through interdisciplinary approaches. Essential for fitness, tractably complex, and relatively well-understood, these interactions thus represent strong study systems for understanding the evolution of adaptive traits. In this article, we present principles and case studies of investigations of the mitonuclear co-evolution of OXPHOS complexes in wildlife. We suggest an integrated experimental approach to this key issue in evolutionary biology, including a novel perspective on the role of genomic architecture in optimizing mitonuclear interactions.

## Oxidative Phosphorylation as an Evolutionary Study System

### OXPHOS Depends on Intimate Mitonuclear Interactions

**FIGURE 1:**
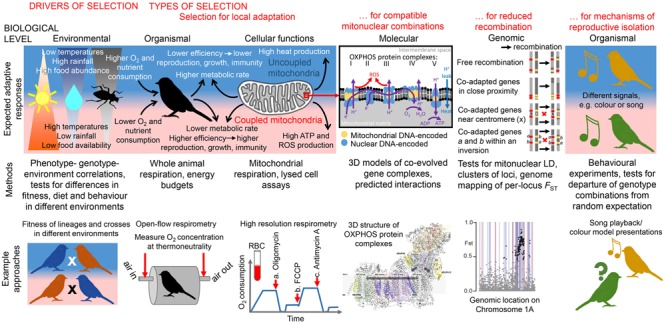
**Overview of expected responses to selective pressures related to thermal metabolism at various levels of biological organization and integrative approaches to studying the mitonuclear interactions they modulate. Environmental level:** Significant differences in temperature and precipitation can drive differences in food abundance and selection for local adaptation. Significant correlations between phenotype, genotype, and environment, after controlling for confounding factors (e.g., genetic drift), can suggest the presence of local adaptation. Fitness/metabolic performance of organisms with diverged mitolineages measured in different environments can indicate the presence of local metabolic adaptation, whereas fitness/metabolic performance of several generations of crosses can show whether mitonuclear incompatibilities have evolved between lineages. **Organismal level:** Heat produced from less-coupled mitochondria may be adaptive in colder environments for endothermic organisms (Pörtner et al., 1998); individuals with less-coupled metabolism are expected to produce fewer ATP molecules (leading to lower amount of energy available for growth, immune function, or reproduction) and fewer ROS (leading to lower oxidative stress and greater longevity) per unit of O_2_/nutrients consumed (Stier et al., 2014a,b). Higher O_2_/nutrient consumption could be expected to compensate for metabolic inefficiency. O_2_ consumption at thermoneutrality can be measured with an open-flow respirometry system (Lighton, 2008) after some acclimation time in a metabolic chamber; this can be used to calculate an organism’s resting metabolism, expected to be lower in organisms adapted to warmer environments (White et al., 2007). **Cellular function level:** The level of mitochondrial coupling between substrate oxidation and ATP production determines the amount of ATP and heat (through proton leak) produced per unit of O_2_/substrate consumed. A low level of coupling resulting in high heat production might be adaptive in cold climates. Mitochondrial respiration in birds, fish, amphibians and reptiles can be measured non-destructively from red blood cells (RBC) instead of liver or muscle tissues; O_2_ consumption (blue line) can be measured at a baseline for comparison with responses to the additions of (a) the ATP synthase inhibitor oligomycin (measures residual O_2_ consumption during proton leakage), (b) the mitochondrial uncoupler FCCP (measures maximal uncoupled O_2_ consumption), and (c) the inhibitor of mitochondrial respiration antimycin A (measures non-mitochondrial oxygen consumption) (Stier et al., 2016). **Molecular level**: Complex I (NADH dehydrogenase), complex II (succinate dehydrogenase), complex III (*bc*_1_ complex) and complex IV (cytochrome *c* oxidase) transport electrons (e^-^) from NADH, succinate and FAD-linked substrates (not shown) to create a proton-motive force (H^+^ gradient). Complex V (ATP synthase) uses energy released by backflow of protons to create ATP from ADP. Proteins of complexes I, III, IV, and V are encoded by both nuclear and mitochondrial genomes, leading to strong selection for compatible functional allele combinations. Mapping genes with amino acid candidates for positive selection onto 3D models of OXPHOS complexes enables better understanding of mitonuclear interactions. Here, the 3D structure of OXPHOS complex I (Fiedorczuk et al., 2016) is shown, with mitochondrially encoded subunits ND4 and ND4L, found to contain positively selected amino acids in two eastern yellow robin lineages (Morales et al., 2016a), highlighted in purple. **Genomic level:** Selection for co-transmission of co-adapted nuclear-encoded mitochondrial allele combinations with mitochondrial DNA lineages can drive the evolution of genomic architecture that suppresses recombination between co-adapted genes; examples of such mechanisms include close proximity of co-adapted genes and location of co-adapted genes near a centromere or within an inversion. Mapping single-nuclear-locus *F*_ST_ between populations fixed for alternative mitochondrial lineages to a reference genome can help detect clusters of loci co-inherited with mtDNA. Here, *F*_ST_s between two eastern yellow robin lineages mapped to chromosome 1A (dots; black dots- top 1% outliers) show the presence of the mtDNA-linked cluster of loci (Morales et al., 2016a); background lines show the location of genes with predicted mitochondrial functions (red lines- OXPHOS genes), for which this genomic region was enriched. **Organismal level (reproductive isolation and incompatibilities):** Selection against incompatible mitonuclear combinations [postzygotic reproductive isolation can drive evolution of prezygotic reproductive isolation and result in speciation (Sloan et al., 2016)]. For example, organisms can advertize their mitonuclear genotypes through differences in color or vocalization (Hill and Johnson, 2013; Hill, 2016). Behavioral experiments involving model presentations can elucidate whether individuals mate assortatively according to their mitonuclear genotype, implying late stages of speciation.

Oxidative phosphorylation depends on the interaction of protein complexes in the inner mitochondrial membrane. In most animals, the four core complexes mediating OXPHOS are encoded by the 13 protein-encoding mitochondrial genes and an estimated 80 nuclear-encoded genes (Nicholls and Ferguson, 2013). The respirasome (comprising complexes I, III, and IV) uses the energy liberated during electron transfer from NADH to O_2_ to drive proton-translocation to the intermembrane space and thus establish a proton gradient across the inner mitochondrial membrane (**Figure 1**) (Gu et al., 2016; Letts et al., 2016; Wu et al., 2016). The ATP synthase (complex V) uses the proton-motive force thus generated to chemiosmotically drive ATP synthesis (Allegretti et al., 2015; Hahn et al., 2016). Nuclear-encoded proteins also serve as electron carriers (e.g., cytochrome *c*), alternative electron inputs (e.g., complex II), and assembly factors (e.g., NDUFC1, SURF1) throughout the OXPHOS system (Mashkevich et al., 1997; Nicholls and Ferguson, 2013; Stroud et al., 2016).

Intimate associations between mitochondrial- and nuclear-encoded subunits are required for the electron transport chain of the respirasome and ATP synthase activity for efficient mitochondrial ‘coupling,’ i.e., the ratio of ATP synthesis per unit substrate and O_2_ consumed (Lowell and Spiegelman, 2000). The intimacy of such interactions is particularly reflected by OXPHOS complex I. This enzyme couples electron transfer through its nuclear-encoded hydrophilic arm to proton translocation through its four mitochondrially encoded proton pumps. This depends on long-range conformational changes mediated through protein–protein interactions of the mitochondrially encoded subunits with core and supernumerary nuclear-encoded subunits (Fiedorczuk et al., 2016; Zhu et al., 2016). Genetic studies have demonstrated that assembly of this complex depends strictly on 39 of its 45 subunits (Stroud et al., 2016), and even single amino acid substitutions can alter coupling efficiency (Mimaki et al., 2012; Gershoni et al., 2014). In addition to serving as the primary electron input in the respiratory chain (Nicholls and Ferguson, 2013), OXPHOS complex I is also the main site of cellular reactive oxygen species (ROS) production (Murphy, 2009).

The central physiological importance of OXPHOS means that mitonuclear compatibilities are required for optimal fitness. Even minor biochemical inefficiencies can have major fitness consequences for an organism by modulating their energetic efficiency and oxidative stress levels. There are therefore strong selective pressures to maintain optimal mitonuclear interactions in the OXPHOS system (Rand et al., 2004; Dowling et al., 2008; Burton et al., 2013).

### Mitonuclear Interactions are Linked to Thermal and Redox Adaptation

There are multiple lines of evidence that OXPHOS is important for local adaptation. Experimental approaches with model organisms have allowed researchers to test an impressive array of mitonuclear combinations and assess their functional effects under a wide set of conditions (Dowling et al., 2007; Arnqvist et al., 2010; Paliwal et al., 2014; Ma et al., 2016; Mossman et al., 2016). These efforts have revealed that mismatched mitonuclear interactions (gene–gene interactions) can have profound consequences, such as reduced metabolic performance, fecundity, and lifespan. When mitonuclear combinations are assessed in multiple environments (e.g., diet, temperature or hypoxia), interaction effects are commonly context dependent (gene–gene-environment interactions; Koevoets et al., 2012; Hoekstra et al., 2013).

In the wild, OXPHOS traits have been correlated with a wide range of environmental pressures, including heat stress (Morales et al., 2016a,b), cold stress (Cheviron et al., 2014; Stier et al., 2014a,b), nutrient limitation (da Fonseca et al., 2008), and hypoxia (da Fonseca et al., 2008; Scott et al., 2011). Consistently, there is evidence for positive selection and climate-linked differences in the sequences and expression of OXPHOS genes in a range of animal species with wide biogeographic ranges (Mishmar et al., 2003; Ruiz-Pesini et al., 2004; Toews and Brelsford, 2012; Garvin et al., 2015; Morales et al., 2016a). Accordingly, genes encoding the OXPHOS machinery are frequently candidates for positive selection. This likely reflects the high levels of mitochondrial DNA variation within and among populations combined with the selection pressures for optimally adapted phenotypes (Gershoni et al., 2009; Bar-Yaacov et al., 2012).

As mechanistic understanding of OXPHOS activity continues to improve, it should be increasingly possible to make more specific predictions of what kinds of protein-level changes should be adaptive. For example, it is hypothesized that the coupling efficiencies of OXPHOS complexes are closely linked to adaptive thermal biology. OXPHOS generates chemical energy and heat in proportions that depend on the coupling efficiency of the respirasome and ATP synthase (Lowell and Spiegelman, 2000). It is proposed that the heat produced from less-coupled mitochondria may be particularly beneficial for adaptation of endothermic organisms to colder environments. In contrast, heat production may be deleterious in warm environments, necessitate higher nutritional intake, and is associated with high oxidative stress due to increased ROS production (**Figure 1**) (Pörtner et al., 1998; Brand, 2000; Somero, 2002; Fangue et al., 2009; Stier et al., 2014a,b). It is important to account for variation in such predictions among organisms and environments. For example, contrary to the expectations for endotherms, cold adaptation in fishes is linked to higher mitochondrial densities in muscle (White et al., 2011), and so the associated high energy demands for synthesis and maintenance of mitochondria may favor genotypes with high coupling efficiency (Pavlova et al., 2017).

### Nuclear Genome Architecture May Facilitate Co-evolution of Mitochondrial-Encoded and Nuclear-Encoded Mitochondrial Genes

Mitonuclear co-evolution should be enforced under strong selection given the complex interactions and essential functions mediated by OXPHOS (Burton et al., 2013). Challenges to positive co-evolution include the fast mutation rate of the mitochondrial genome (due to its proximity to ROS production, high rate of replication and lack of efficient repair mechanisms), typically maternal inheritance, and lack of recombination, which generate a mutation load that the nuclear genome must counter by compensatory mutation (Rand et al., 2004; Lynch et al., 2006; Osada and Akashi, 2012; Havird et al., 2015; Havird and Sloan, 2016). In addition, mitonuclear co-evolution can be disrupted by mechanisms that generate genetic variation or promote gene flow. In particular, substantial gene flow and recombination in nuclear DNA will tend to break up optimally functioning allele combinations of co-adapted genes in each sexual generation (Rand et al., 2004; Burton and Barreto, 2012; Burton et al., 2013). Accordingly, we propose that nuclear genomic architecture should tend to evolve to suppress recombination and prevent the segregation of genome regions that mediate epistatic functions of nuclear-encoded mitochondrial genes.

With improvements in techniques to explore genome structure, examples are building of how genomic architecture can drive evolutionary adaptation, for example the ‘supergene’ of 125 genes associated with differences in male mating strategies in birds (Küpper et al., 2016). Natural selection can locate co-adapted loci in genome areas of low recombination (Schwander et al., 2014; Thompson and Jiggins, 2014) or promote genomic clustering of synergistically adaptive alleles (Yeaman, 2013) so that they can be co-inherited and/or co-regulated. The three-dimensional organization of the genome can dictate how and which loci should be subject to genome changes that will favor their co-location (Lanctôt et al., 2007; Wijchers and de Laat, 2011). Reduced recombination among co-adapted genes (increasing their co-inheritance) can occur through the evolution of recombination modifiers or chromosomal re-arrangements, such as transposition of a gene to a location close to a co-adapted gene, movement of co-adapted genes toward a centromere or into a region within an inversion between diverged lineages (**Figure 1**) (Rieseberg, 2001; Butlin, 2005; Kirkpatrick and Barton, 2006; Yeaman and Whitlock, 2011; Yeaman, 2013; Ortiz-Barrientos et al., 2016).

To date, mitonuclear genomic architecture (encompassing mitochondrial-nuclear and nuclear-nuclear) remains relatively underexplored, except in the context of biased co-transmission of mitochondrial and nuclear-encoded mitochondrial genes on sex chromosomes. Because mtDNA, due to its maternal inheritance, accumulates mutations that are deleterious in males (mother’s curse), selection to restore fitness in males that drives compensatory evolution of nuclear-encoded mitochondrial genes could be expected to prevent concentrations of such genes on female-linked chromosomes (Havird and Sloan, 2016). Results for different taxa variously support overrepresentation, underrepresentation, or unbiased distribution of nuclear-encoded mitochondrial genes on X and Z chromosomes with respect to autosomes, supporting multiple theories of the distribution of nuclear-encoded mitochondrial genes: co-adaptation, sexual conflict and sexual selection (Drown et al., 2012; Hill and Johnson, 2013; Dean et al., 2014, 2015; Hill, 2014; Rogell et al., 2014). More recently, we uncovered an autosomal genomic island of divergence associated with mitonuclear interactions in a passerine (**Figure 1**) (Morales et al., 2016a). This genomic island of divergence is implicated in maintaining deep mitochondrial divergence between two parapatric lineages connected by nuclear gene flow. Observations of the fluidity of positioning of nuclear-encoded mitochondrial genes in some systems (but see Dean et al., 2015) raise questions about the role of genome organization in rates of co-evolution between mitochondria and nuclear genes (Hill, 2014). We contend that exploration of genomic architecture may be crucial for understanding mitonuclear co-evolution, and also vice versa given the crucial roles and considerable number of genes concerned with mitochondrial function (Pagliarini et al., 2008).

## Integrative Approaches for Studying Mitonuclear Co-Evolution

While signals of selection have frequently been identified in OXPHOS-encoded genes, few studies have examined changes in function due to observed substitutions; thus empirical demonstration of local adaptation is limited (Burton et al., 2013; Levin et al., 2014). However, it is possible to develop relatively strong genotype-to-phenotype links by bridging population genetic studies with biochemical and physiological approaches developed for studying OXPHOS. In the five decades since its discovery by Mitchell (1961), a wealth of physiological, biochemical, and structural studies have developed a rich understanding of oxidative phosphorylation (Nicholls and Ferguson, 2013), and much of the knowledge and methodology can be translated to wild populations.

Here we suggest a flexible framework that draws on recent technical advances in multiple fields for testing the significance of mitonuclear interactions. First, candidate interacting loci can be identified by improved methods for inferring loci under selection. Second, these candidates can then be examined through structural mapping and modeling to develop hypotheses about biochemical interactions relevant to the species biology in question. Third, these hypotheses can then be tested by measuring phenotypic responses at different scales, notably whole cell, whole animal, and fitness in the wild. Finally, experimental approaches could be used to test for reproductive isolation between differently adapted lineages (**Figure 1**). It is particularly desirable to compare multiple species for repeated signals of selection in the same genomic regions: common signals of selection between lineages in the context of their geographic arrangement relative to selection pressures provide strong evidence of adaptation (Garvin et al., 2014). Adopting this proposed framework should increase comparability among studies.

### Genetic Approaches for Detecting Natural Selection

Detection of natural selection is one of the most contentious and active fields in evolutionary biology. Here we highlight, in the context of mitonuclear co-evolution, the more general issues that are explored in depth elsewhere (Nielsen, 2005; Haasl and Payseur, 2016; Manel et al., 2016; Stephan, 2016).

The power to detect candidate loci that evolve under natural selection rests on the molecular tools available for a given system: reduced representation genomic scans (SNPs), sequence-based genomic scans (candidate genes, exome-sequencing, or RNA-sequencing), whole genome re-sequencing, and/or physical linkage maps (Manel et al., 2016; Stephan, 2016). The key limitation to detecting natural selection in the wild is that several ecological and evolutionary processes can leave a similar signature to selection and lead to a high rate of false positives. Confounding factors include demographic processes (e.g., population size change and structure), background selection, and heterogeneous mutation and recombination rates. Given that mitochondrial and nuclear genomes can have largely independent evolutionary histories (e.g., different introgression patterns and mutation load), knowledge of the demographic history of the study system is especially useful to interpret patterns of mitonuclear co-evolution (Bar-Yaacov et al., 2015; Morales et al., 2016a,b; Pereira et al., 2016; Sloan et al., 2016). Given that mitonuclear co-evolution is likely to respond to environmental variation (Burton et al., 2013), approaches to detecting selection that rely on gene-environment associations could be particularly useful to identify candidate loci under selection (Rellstab et al., 2015; Forester et al., 2016).

A common starting point in the search for signatures of selection in mitonuclear co-evolution is sequencing full mitochondrial genomes. A family of methods proven to be especially useful in the context of mitogenome evolution are codon-based approaches, which rely on the estimation of the non-synonymous to synonymous ratio (ω = *d*_N_/*d*_S_) [HyPhy and Datamonkey (Pond and Frost, 2005); PAML (Yang, 2007)]. There are multiple examples in the literature of how complementary codon-based approaches have been combined to discriminate positive and relaxed purifying selection in mitogenome-encoded OXPHOS components (da Fonseca et al., 2008; Garvin et al., 2011; Morales et al., 2015; Wertheim et al., 2015; Pavlova et al., 2017). However, these types of methods have important limitations: they require data across multiple species, or sequences that are reasonably diverged, and only allow selection inference within coding regions (Gonçalves da Silva, 2017). It is important to consider this last limitation since mitonuclear incompatibilities have been mapped to non-coding regulatory genes, non-coding sequences such as transfer RNAs and the mitochondrial control region (Montooth et al., 2009; Meiklejohn et al., 2013; Rollins et al., 2016; Jhuang et al., 2017).

A natural follow-up is to look for evidence of natural selection in nuclear-encoded mitochondrial genes and signals of mitonuclear co-evolutionary adaptation (Mishmar et al., 2006; Gagnaire et al., 2012; Bar-Yaacov et al., 2015; Pereira et al., 2016). For example, such approaches have identified supernumerary and assembly factors of OXPHOS complex I implicated in local adaptation (Garvin et al., 2016; Morales et al., 2016a).

### Protein Mapping and Modeling Enable Development of Mechanistic Hypotheses

Recent advances in understanding structure-function relationships in oxidative phosphorylation enable better prediction of how genetic substitutions affect mitochondrial function. Largely as a result of recent advances in cryo-electron microscopy, complete atomic-resolution structures of all components in the mammalian electron transport chain are now available, including the mitochondrially co-encoded complex I (**Figure 1**) (Fiedorczuk et al., 2016; Zhu et al., 2016), complex III (Iwata et al., 1998), complex IV (Tsukihara et al., 1996), and the respirasome supercomplex (Gu et al., 2016; Letts et al., 2016; Wu et al., 2016). In addition, near-complete structures of yeast ATP synthase are also available (Allegretti et al., 2015; Hahn et al., 2016).

With these newly available protein structures, it is now possible to map the locations of subunits and amino acids predicted to be under selection using protein visualization software (Pettersen et al., 2004) and to develop homology models using public servers (Källberg et al., 2012; Kelley et al., 2015). Such approaches have been used to predict the mechanistic effects of amino acid substitutions observed in OXPHOS subunits across diverse species (Scott et al., 2011; Finch et al., 2014; Caballero et al., 2015; Zhang and Broughton, 2015; Campana et al., 2016; Morales et al., 2016a). This approach was first highlighted by Garvin et al. (2011) who detected an amino acid under positive selection in the Pacific salmon in an unusual region of OXPHOS complex I: the piston-like horizontal helix (helix HL) of ND5. Meta-analysis has since suggested that this helix is the most common region of positive selection in the mitogenomes of diverse animal species (Garvin et al., 2014). While the function of the helix HL remains unresolved, it is hypothesized to influence coupling by propagating conformational changes from proximal to distal proton pumps; hence fine-tuning its properties may have adaptive consequences for heat and energy production (Torres-Bacete et al., 2011; Sazanov, 2014).

### Bridging Gaps through Mitochondrial, Cellular, and Organismal Physiology

In animal systems, it is challenging to validate experimentally that certain amino acid substitutions affect mitochondrial function. Due to their membrane localisation, multi-subunit cofactor-bound composition, and complex assembly pathways, OXPHOS complexes are incompatible with recombinant protein expression and can rarely be purified natively (Nicholls and Ferguson, 2013). However, a suite of physiological techniques enable us to measure the activities, kinetics, and efficiencies of OXPHOS complexes. For example, classical respirometry techniques enable measurement of the rates of substrate oxidation or oxygen consumption by whole cells or purified mitochondria; it is possible to calculate mitochondrial coupling efficiencies and to probe the activities of specific protein complexes by systematically comparing basal respiration rates with those in the presence of specific agonists, inhibitors, and uncouplers (**Figure 1**) (Nicholls and Ferguson, 2013; Stier et al., 2013, 2016; Toews et al., 2014). Well-established assays using lysed cells also enable measurement of potentially relevant parameters such as the expression levels, protein content, and kinetic parameters of individual OXPHOS complexes (Scott et al., 2011).

Another development that enhances the ability to measure biologically relevant mitochondrial function is the recent discovery that non-mammalian animals harbor functional mitochondria in their erythrocytes. This presents options for non-destructive sampling of wild populations (Stier et al., 2013, 2015, 2016). These cellular measurements of mitochondrial respiration can be complemented with whole-organism measurements of basal and maximal metabolic rates; while rarely adopted in mitonuclear ecology, such approaches may have value for understanding relationships between nutritional intake and energy expenditure (White et al., 2007, 2011; Halsey and White, 2010).

Taking an approach amenable to experimental manipulations, laboratory-based crossings have also been used to assess the effects of intraspecific and interspecific mitonuclear compatibilities using individuals sampled from wild populations from different environments. As elaborated in a case study below, there are several examples of how crosses have been combined with measurements of enzymatic, cellular, or organismal performance to consolidate genotype-to-phenotype links (Edmands and Burton, 1999; Willett and Burton, 2001; Arnqvist et al., 2010; Chang et al., 2015; Dordevic et al., 2016). In the few study systems where cell lines can be established, experimental cellular approaches can provide valuable functional insights in study systems (Blier et al., 2006), for example through the construction of mitonuclear hybrid cell systems (cybrids) that allow testing of the effects of mitogenome variation on fitness in a constant nuclear background (e.g., Barrientos et al., 1998; Moreno-Loshuertos et al., 2006; Dingley et al., 2014). Within these kinds of manipulative approaches, as well as more broadly, rapid advances in molecular genomics, including the increasing tractability of RNA sequencing, are facilitating investigations of the roles, mechanisms and evolutionary genomics of genes of interest in adaptation and divergence of wild species (Harrisson et al., 2014; Havird and Sloan, 2016; Latorre-Pellicer et al., 2016).

### Gaining Insights into the Genomic Architecture of Mitonuclear Co-adaptation

The level of resolution that can be reached in examining genomic architecture depends on the genomic resources available. Full resolution requires assembled genomes and physical linkage maps, rarely available for wild organisms. However, powerful approximations can be made by mapping genetic variants of interest (e.g., candidate genes or loci under selection) on to a reference annotated genome of the same species or a close relative with known conserved synteny. Mapped variation provides the presumed order, position and identity of loci of interest (e.g., nuclear-encoded mitochondrial genes).

Linkage (gametic) disequilibrium (LD) is the non-random association of alleles at different loci within individuals. These correlations can arise through genes being near each other on a chromosome, via population subdivision, and driven by epistatic selection. Accordingly, LD can arise between markers on different nuclear chromosomes, and between the mitochondrial and nuclear genomes (Sloan et al., 2015). Linkage disequilibrium has proven a powerful tool for studying the genomic architecture of population divergence, local adaptation, and reproductive isolation (Nosil et al., 2009; Servedio, 2009; Smadja and Butlin, 2011). Natural selection can favor the evolution of high LD when multiple loci that influence a trait experience the same divergent selection (Nielsen, 2005). Strong LD between nuclear-encoded mitochondrial alleles could signal co-adapted genes responding to the same selective drivers, which may or may not be maintained by genomic architecture favoring reduced levels of recombination. Significant clustering of nuclear genes encoding mitochondrial or chloroplast proteins in *Arabidopsis* has been demonstrated (Alexeyenko et al., 2006). Similar analysis for animal taxa has rarely been performed, not least because fully assembled genomes are unavailable for many of the organisms for which mitonuclear co-evolution might be relevant, but some significant mitochondrial-nuclear LD is present in humans (Sloan et al., 2015). As genomic resources for non-model organisms expand, we expect to see more studies of mitonuclear genomic architecture (e.g., genomic re-arrangements of nuclear-encoded mitochondrial genes), as is increasingly the case for other traits linked to reproductive isolation (Lowry and Willis, 2010; Jones et al., 2012; Egan et al., 2015).

Even without a reference genome, LD can be estimated by analyzing LD clustering with LDna (Kemppainen et al., 2015). This tool finds clusters of loci with similarly high levels of LD independently of their position in the genome. This is a valuable first step to studying the genomic architecture of organisms without genomic resources. It is important to note that high LD can also emerge through processes unrelated to selection, such as population history and structure, which should be accounted for (Mangin et al., 2012; Goicoechea et al., 2015). Demographic factors, however, should impact many loci across the genome, so significant excesses of high LD among nuclear genes with mitochondrial functions is indicative of non-neutral processes (Morales et al., 2016a). Comparative genomic approaches are also recommended to investigate whether re-arranged or ancestral genomic architectures in closely related taxa are more or less prone to evolution of mitonuclear interactions, and whether nuclear-encoded mitochondrial gene re-arrangements can be favored to reduce recombination between locally co-adapted alleles and can promote adaptive genetic divergence (Faria et al., 2011; Yeaman, 2013).

## Case Studies Highlighting Integrative Approaches

The strength of oxidative phosphorylation as an evolutionary study system has been highlighted by several in-depth studies on specific organisms. Each of these studies integrated different combinations of the techniques described above to address different questions about the role of OXPHOS in evolutionary and ecological processes.

### Mitochondrial Selection in High-Altitude Adaptation in Bar-Headed Geese

One of the richest examples of the role of mitochondrial evolution in local adaptation comes from studies of the bar-headed goose *Anser indicus*. During its well-documented migrations over the Himalayas, this bird sustains high metabolic rates as a result of a multitude of physiological adaptations (Bishop et al., 2015). Comparative physiological studies show that adaptations at multiple levels of organization, from protein activity to organ morphology, enable this species to enhance O_2_ supply and modulate O_2_ usage compared to low-altitude geese species (Scott et al., 2009, 2011).

Among the potentially adaptive differences observed is a difference in the substrate kinetics of OXPHOS complex IV in bar-headed geese compared to other species. Cardiac muscle measurements show that this enzyme has a comparatively high affinity but low activity for its nuclear-encoded substrate cytochrome *c*. Scott et al. (2011) proposed that this may be adaptively relevant by enabling the mitochondrion to maintain redox balance in response to limitations and fluctuations in their O_2_ supply during their extreme flights. The authors inferred the genotypic basis of these changes by comparing the sequences of the mtDNA and nuclear-encoded mitochondrial genes from complex IV subunits between low- and high-altitude species. This revealed several non-synonymous substitutions in bar-headed geese, including a unique W116R substitution in COX3, as well as subtle differences in the expression levels of the complex (Scott et al., 2011).

Despite these strong phenotype-to-genotype links, it remains to be determined how these substitutions affect the biochemistry of the complex. Homology modeling suggests that the W116R mutation disrupts inter-subunit interactions in complex IV, but it is unclear if and how this affects the binding of cytochrome *c* (Scott et al., 2011). These gaps reflect the major challenges associated with purifying this enzyme complex for kinetic or structural characterisation. A striking contrast is provided by studies on why the O_2_-affinity of hemoglobin is so high in bar-headed geese; the relative ease of purifying this protein from erythrocytes has facilitated structural studies showing that a single amino acid substitution markedly shifts the cooperative behavior of the hemoglobin tetramer and in turn modulates O_2_ affinity (Zhang et al., 1996; Liang et al., 2001).

These studies of the bar-headed goose offer a strong example of integrated research leading to an understanding of the mechanistic links between genes affecting mitochondrial function and ecophysiological phenotype. While there are multiple classes of gene that might be expected to contribute to the adaptive phenotype (Scott et al., 2011), there is no particular expectation that strong genome architecture is required to promote the co-evolution of these: the species does not interbreed with another, and there are no apparent differently adapted lineages within the bar-headed goose, so gene flow should not disrupt co-adapted combinations.

### Mitonuclear Co-evolution in the Marine Crustacean *Tigriopus californicus*

Through extensive studies, Burton and colleagues have demonstrated that mitonuclear discordance has contributed to allopatric population divergence of *Tigriopus californicus* copepods. There is extraordinary genetic differentiation between populations of this intertidal copepod across geographic barriers in the Californian coast, with mtDNA divergence exceeding 18% between Santa Cruz and San Diego populations (Burton and Lee, 1994; Burton, 1998). Elegantly designed aquarium experiments revealed that F_1_ hybrids from the *T. californicus* populations are viable, but subsequent generations exhibit a range of fitness defects and reduced ATP production rates (Burton and Lee, 1994; Ellison and Burton, 2006, 2008). Consistent with a mitochondrial origin, maternal but not paternal backcrossing can restore fitness of progeny (Ellison and Burton, 2008).

Targeted sequencing revealed that there are high levels of divergence in genes encoding key determinants of the mitochondrial electron transport chain. While most of these substitutions appear to be neutral, ω-based approaches provided strong evidence for positive selection for substitutions in mitochondrially encoded complex IV and its nuclear-encoded substrate cytochrome *c* (Rawson et al., 2000; Willett and Burton, 2004). Interpopulation crossing experiments substantiated this by showing that mitonuclear compatibility was required for optimal complex IV activity (Edmands and Burton, 1999) and that the mitotype modulated segregation ratios of cytochrome *c* (Willett and Burton, 2001). The authors went further by validating these predictions using biochemical approaches. Intrapopulation pairs of complex IV and cytochrome *c* consistently showed up to fourfold higher activity than did interpopulation pairs (Rawson and Burton, 2002). Moreover, recombinantly produced cytochrome *c* variants (reflecting different nuclear backgrounds) interacted differentially with complex IV in tissue homogenates (reflecting different mitochondrial backgrounds) (Harrison and Burton, 2006). This work proved that single substitutions are sufficient to cause mitonuclear incompatibilities in wild populations.

Despite these accomplishments, the evolutionary processes and pressures that result in allopatric population divergence remain under investigation. Pereira et al. (2016) recently approximated the contribution of genetic drift and natural selection in *T. californicus* divergence by comparing whole-transcriptome sequences of allopatric populations at different stages of divergence (Pereira et al., 2016). They found that the pattern of shared polymorphism could be partially explained by genetic drift, as lower effective population sizes led to less shared polymorphism between populations, and higher mutation load. However, natural selection possibly drives accelerated evolution of some genes, including nuclear-encoded mitochondrial ones. The authors predict that genomic architecture should regulate the efficiency of selection and the impact of drift, for example by modulating recombination rates, but this prediction was not tested.

### Genomic Architecture of Mitonuclear Interactions in the Eastern Yellow Robin

Our studies on the population structure of eastern yellow robin *Eopsaltria australis* have emphasized the importance of studying genomic architecture (Morales et al., 2016a). This songbird is one of multiple animals that maintains functional mitonuclear interactions despite discordance between its mitochondrial and nuclear genomes (Toews and Brelsford, 2012). Population genetic data have shown that the major axis of nuclear DNA differentiation runs north-south through the species range in Eastern Australia, whereas mitochondrial DNA has diverged into two mitolineages in the perpendicular coastal-inland direction (Pavlova et al., 2013; Morales et al., 2016a). Coalescent analyses suggest that the two genomes initially differentiated together in a north–south direction during the early Pleistocene, but their evolutionary history became separated due to two mitochondrial introgression events in the mid-to-late Pleistocene (Pavlova et al., 2013; Morales et al., 2016b). The two mitolineages show sharp climate-correlated differences in their distributions. This suggests that the mitochondrial introgression and resulting divergence were driven by natural selection (Morales et al., 2016b).

There is evidence of positive selection for non-conservative amino acid differences in the proton pumps ND4 and ND4L of OXPHOS complex I between the mitolineages of the eastern yellow robin. These polymorphisms are predicted to cause differences in electrostatic subunit-subunit interactions and in turn influence coupling efficiencies of the complex, though this remains to be validated experimentally (Morales et al., 2015). Comparison of fixation indexes in the nuclear genomes between eastern yellow robin populations across their biogeographic range revealed the existence of two genomic islands of divergence against a background of low differentiation (Morales et al., 2016a). One of these islands, located on autosome 1A (**Figure 1**), is statistically overrepresented with nuclear-encoded genes with predicted mitochondrial functions; among them are three complex I supernumerary subunits proposed to be functionally linked to the mitochondrially encoded ND4 and ND4L genes. Moreover, markers within the genomic island of divergence exhibit very strongly elevated LD, suggesting genome architecture that promotes reduced recombination between nuclear-encoded genes with mitochondrial functions. Further research will disentangle whether mitonuclear co-evolution promoted the evolution of this particular genomic architecture or pre-existing genomic architecture enabled this tight mitonuclear co-evolution.

## Conclusion

In this perspective, we have summarized some of the wealth of information of the adaptive consequences of mitochondrial-nuclear interactions, with particular focus on OXPHOS functions. We make the case that the powerful fitness consequences of mitonuclear gene interactions are likely to select for optimized genome architecture that will hold together effective combinations of nuclear-encoded mitochondrial gene in the face of gene flow. Demonstrating the phenotypic consequences of genome variation in wildlife species is challenging: we suggest a workflow that utilizes advances in detection of candidates of selection, biochemical understanding of OXPHOS, phenotyping and studying genome organization. The number of studies demonstrating major evolutionary impacts of mitonuclear interactions in wildlife is currently limited, but we anticipate they will be found to be common under the application of the strong emerging methods of investigation such as we present here. Comparative genomic approaches will be important for deriving the deepest insights into mitonuclear evolution and genome architecture, and accordingly, we encourage the application of consistent methodologies.

## Author Contributions

All authors listed have made substantial, direct and intellectual contribution to the work, and approved it for publication.

## Conflict of Interest Statement

The authors declare that the research was conducted in the absence of any commercial or financial relationships that could be construed as a potential conflict of interest.
